# Rare presentation of multiple thromboses in Erdheim–Chester disease: a case-based review

**DOI:** 10.1007/s12185-022-03393-x

**Published:** 2022-06-10

**Authors:** Yongchang Liu, Changpin Huang, Xiaohu Meng, Xin Fang, Xupin Xie, Changrong Wang, Meiyun Wang

**Affiliations:** 1 Department of Vascular Surgery, Affiliated Hangzhou First People’s Hospital, ZheJiang University School of Medicine, Zhejiang Province, Hangzhou, 310006 China; 2 Department of General Surgery, Hangzhou Geriatrics Hospital, Zhejiang Province, Hangzhou, 31006 China; 3 Department of Pathology, The First Affiliated Hospital, School of Medicine, Zhejiang University, Hangzhou, China; 4 Department of Oncology, The First People’s Hospital of Xiaoshan District, 199# Shixin RdZhejiang Province, Hangzhou, 311200 China

**Keywords:** Bone lesion, Coated aorta, Erdheim-Chester disease, IL-6 antagonist, Thrombosis

## Abstract

**Background:**

ECD is a rare non-Langerhans cell histiocytosis with diverse and heterogeneous clinical manifestations, ranging from single-lesion forms to multi-system involvement, including slowly progressing unifocal forms to rapidly evolving life-threatening disease.

**Case presentation:**

A female patient presented with a 2-month history of fever. Imaging revealed multiple thromboses, bone destruction, an abnormal pituitary stalk, and clinical manifestations of diabetes insipidus. Excisional biopsy of a tibial lesion was sent for microscopic examination, and subsequent immunohistochemical testing was positive for expression of CD68 and CD163, and negative for expression of the immune markers CD1a, S100, and langerin. This confirmed the diagnosis of ECD. Treatment with methylprednisolone to inhibit the immune inflammatory response along with anti-cytokine therapy with an interleukin-6 antagonist resulted in satisfactory disease control.

**Conclusion:**

We report a rare case of multiple thromboses, embolism, and multiple organ involvement as the main presentation of ECD, suggesting that ECD should be considered in patients presenting with multiple thromboses associated with multisystem damage. We successfully treated our patient with glucocorticoids and interleukin-6 antagonist. This patient’s response to treatment suggests that hormone therapy and cytokine/chemokine therapy may be a potential novel treatment for patients with ECD without gene mutations.

## Introduction

Erdheim–Chester disease (ECD) is a rare non-Langerhans cell histiocytosis characterized by chronic uncontrolled inflammation and organ infiltration by CD68(+), CD1a(−) non-Langerhans foamy histiocytes, surrounded by fibrosis [1, 2]. Previous studies have revealed that the disease is driven by mutations in proto-oncogenes such as *BRAF* and *MEK*, while immune-mediated mechanisms also contribute to disease development and progression. The manifestations may thus mimic those of other neoplastic and systemic immune-mediated diseases [3]. The origin of ECD is currently unknown and its clinical manifestations are diverse and highly heterogeneous, ranging from single-lesion forms to multiple-system involvement, and including slowly progressing unifocal forms to rapidly evolving life-threatening disease. Almost any organ can be involved, but the most common sites include the long bones, retroperitoneal fibrosis, interstitial lung disease, pericardial and myocardial infiltration, the central nervous system (CNS), the retro-orbital region, and large-vessel involvement [4–6]. The lack of specific manifestations and markers and its difficult differential diagnosis thus present challenges. Cardiovascular involvement is common in ECD, with 40–42% of patients having cardiovascular disease, noted either by multimodal imaging or by medical history taking [7, 8]. However, vascular involvement is rare, and we found a few previous reports of polythrombosis in patients with ECD. Here, we present an unusual case of a patient with ECD characterized by multiple thromboses causing organ embolism as the main clinical manifestations. This case involved multiple systems with complex clinical manifestations and was investigated by laboratory examinations, imaging, pathology, immunohistochemistry, and genetic testing, and treated with systemic and local treatments and monitoring. We describe the detailed characteristics of ECD in this case, with the aim of improving its recognition and differential diagnosis.

## Case report

A 36-year-old postpartum Chinese female (1 month) patient was referred to our hospital with a 2-month history of fever and abdominal colic, which showed a gradual onset and low-to-moderate intensity sharp pain. She also presented with pain in her right shoulder and bilateral distal interphalangeal joints, and with polyuria.

The patient had a history of prenatal infertility and was conceived by in vitro fertilization. The examination results related to the disease history were normal. She was otherwise healthy, with no other medical history.

One month previously, the patient had developed long-term, mainly moderate/high fever. Blood cultures sent for multiple tests were negative. Her temperature remained high despite the repeated application of various potent broad-spectrum antibiotics. Diabetes insipidus was diagnosed based on a urine volume >5000 mL/day.

**Fig. 1:**
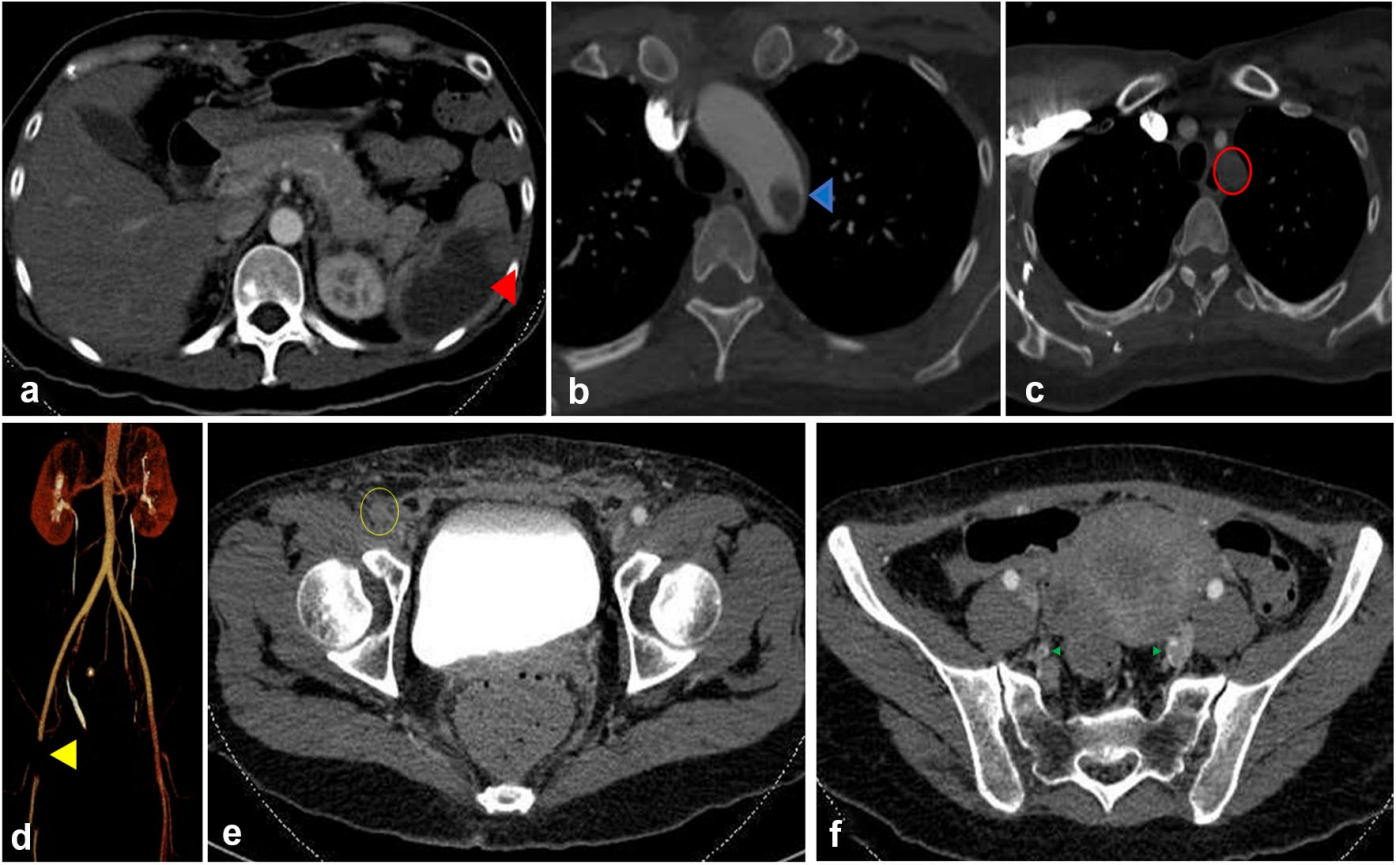
Computed tomography angiography images. **a** Spleen infarction (red triangle). **b** Thrombosis of the thoracic aorta (blue triangle). **c** Subclavian artery thrombosis (red circle). **d** Left common femoral artery embolism (yellow triangle). **e** Left common femoral artery embolism (yellow circle). **f** Bilateral internal iliac artery embolism (green triangle)

Laboratory analysis showed normal levels of rheumatoid factor, antinuclear antibodies, antibody against cyclocitrullin, antineutrophil cytoplasmic antibody, anticardiolipin antibody, anti-beta2 glycoprotein a, i-kinetochore antibody, alexin, uric acid, and antistreptolysin O. Her complete blood count suggested anemia (hemoglobin 68 g/L, red blood cells 2.49 × 10^9^), low IgG (5.96 g/L) and IgM (0.39 g/L), and high levels of C-reactive protein (CRP, 60.2 mg/L), a high erythrocyte sedimentation rate (ESR, 36 mm/h), and high interleukin-(IL) 6 (198.69 pg/mL). Her metabolic panel, including liver function tests, was normal except for albumin (20.5 g/L), sodium (165 mmol/L), and chloride (127 mmol/L). Her coagulation function was normal except for D-dimer (24,920 µg/L). Her protein S was normal; her protein C (68.7%, 70.0–140.0) and antithrombin III were slightly lower (65.8%, 75–125); her coagulation factors II, VII, and IX were normal, and factor VIII (219.3%, 50–150) was elevated. Her total calcium (1.7 mmol/L), serum 25-hydroxy-vitamin D (1083 pg/mL), parathyroid hormone (34.89 pg/mL), and phosphorus (1.3 mmol/L) were determined. Bone marrow biopsy of the posterior superior iliac spine showed slightly low hematopoietic hyperplasia, granulocyte hyperplasia, and mainly mid and late myelocytic hyperplasia; the granulocyte/erythrocyte ratio was normal, and 5–10 megakaryocytes/LPF were observed. The immunohistochemical results were as follows: S100 (−), CD1a (−), CD6, CD8 (+), Langerin (−), CD163 (+), MPO (+), and CD71 (+). Abdominal computed tomography (CT) revealed a splenic infarction (Fig. 1a). The mesentery and omentum space were opaque and showed multiple small exudative lymph nodes. Thoracic aortic CT angiography (CTA) revealed multiple thromboses of the aortic arch (Fig. 1b) and left subclavian artery (Fig. 1c). Lower limb artery CTA revealed thromboses of the right side of the common femoral artery (Fig. 1d, e), and bilateral internal iliac arteries (Fig. 1f).

**Fig. 2:**
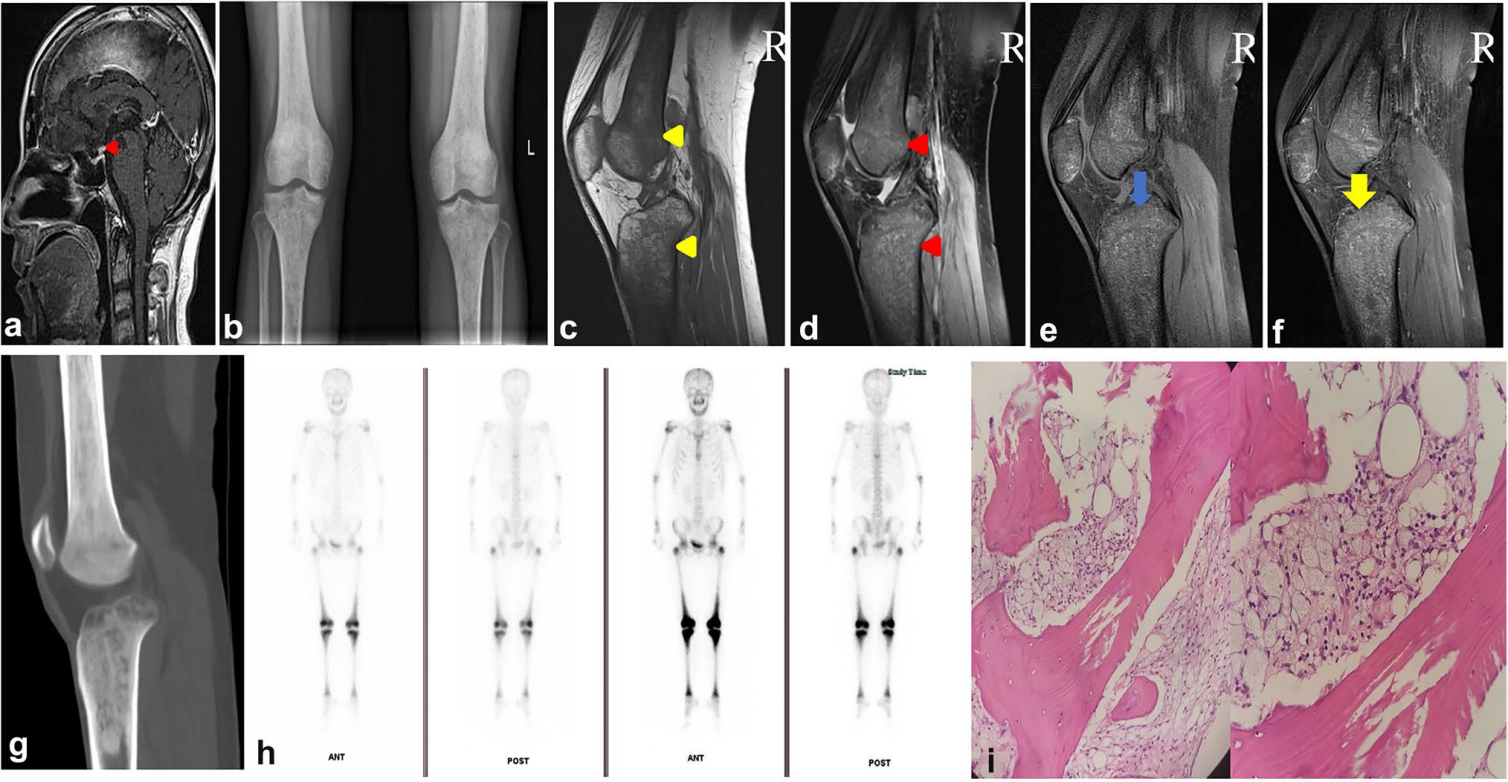
Magnetic resonance imaging (MRI), computed tomography (CT), and X-ray images. **a** MRI of enlarged pituitary stalk (red triangle). **b** Anteroposterior radiographs of bilateral knee joints showing extensive bone destruction of bilateral upper tibia and lower femur. **c** MRI showing long T1 signal of lower femur and upper tibia (yellow triangle). **d** MRI of lower femur and upper tibia showing long T2 signal of fat compression (red triangle). **e** MRI showing fat suppression (blue arrow). **f** MRI enhancement scan showing obvious enhancement (yellow arrow). **g** CT of right knee showing high-density image in bone marrow cavity. **h** ^99m^Tc-methylene diphosphonate bone scans showing enhanced metabolism in the 8th thoracic vertebra, bilateral shoulder joints, bilateral greater trochanter of the femur, bilateral middle and lower segments of the femur, and bilateral tibia ends. **i** Hematoxylin and eosin staining. Magnification ×200 and ×400. Right tibia biopsy showing abundant foam cells and a few multinucleated giant cells in the bone marrow tissue, and adhesive lines in the bone trabeculae

Magnetic resonance imaging (MRI) including the pituitary revealed an eosinophilic granuloma in the pituitary stalk (Fig. 2a). B-mode ultrasound of the lymph nodes revealed multiple enlarged lymph nodes in the neck and armpit. The findings were confirmed by fluorodeoxyglucose positron emission tomography, which showed polyosteopathy, multiple lymph node enlargement, and lipid infiltration. After stenting of the multiple thromboses, high glucose uptake was observed in the right atrium and pituitary stalk. Knee X-ray (Fig. 2b) showed extensive bone abnormalities in both tibias and the femur bone. MRI of the right knee showed multiple focal bone marrow lesions that appeared hyperintense on proton density fat-saturated images (Fig. 2c–f) and hypointense on T1-weighted imaging of the femurs and tibias. We also detected diffuse bone changes in the right knee joint and high-density bone marrow in the lumens of the femur and tibia on three-dimensional CT of the right knee (Fig. 2g). ^99m^Tc-methylene diphosphonate bone scans showed uptake in the 8th thoracic vertebra, bilateral shoulder joints, bilateral greater trochanter of the femur, bilateral middle and lower segments of the femur, and bilateral tibia ends (Fig. 2h).

Considering the life-threatening multiple thromboses, we carried out emergency stent implantation in the thoracic aorta and left common carotid artery, embolectomy of the superficial femoral artery, popliteal artery, posterior tibial artery, and anterior tibial artery, and balloon dilatation of the popliteal artery. We also considered the possibility of in situ thrombosis or derived thrombus in other places and could not rule out the possibility of infection. We therefore tested for thrombotic pathology, which was diagnosed as s thrombus. Bacterial and viral infections were therefore ruled out.

Based on the above patient’s history and related examinations, we diagnosed ECD. Microscopic examination of a tibia lesion showed infiltration of bony tissue with abundant foamy histiocytes surrounding fibrotic proliferation and few multinucleated giant cells (Fig. 2i). Subsequent immunohistochemical analysis was positive for CD68 and CD163 and negative for the immune markers CD1a, S100, and langerin.

Given the role of *BRAF* mutations as the main driver of treatment decisions, we examined relevant gene mutations in this patient but all were negative, including *BRAF*V600E and *MAP2K1*. The patient was also negative for the combined detection of the following *KRAS/NRAS* gene mutations: *KRAS* exon 2 mutations G12S/G12D and G12C/G12R/G12V/G12A/G13D; *KRAS* exon 3 mutations A59T/Q61K and Q61L/Q61R/Q61H; *KRAS* exon 4 mutation K117N/A146T/A146V/A146P; *NRAS* exon 2 mutations G12S/G12D, G12C G12V/G12A G13R/G13V and G13D; *NRAS* exon 3 mutation Q61K/Q61L/Q61R/Q61H; and *NRAS* exon 4 mutation A146T; and for the following five *PIK3CA* mutations: H1047R, H1047L, E542K, E545K, and E545D. JAK2 mutation was negative.

**Fig. 3:**
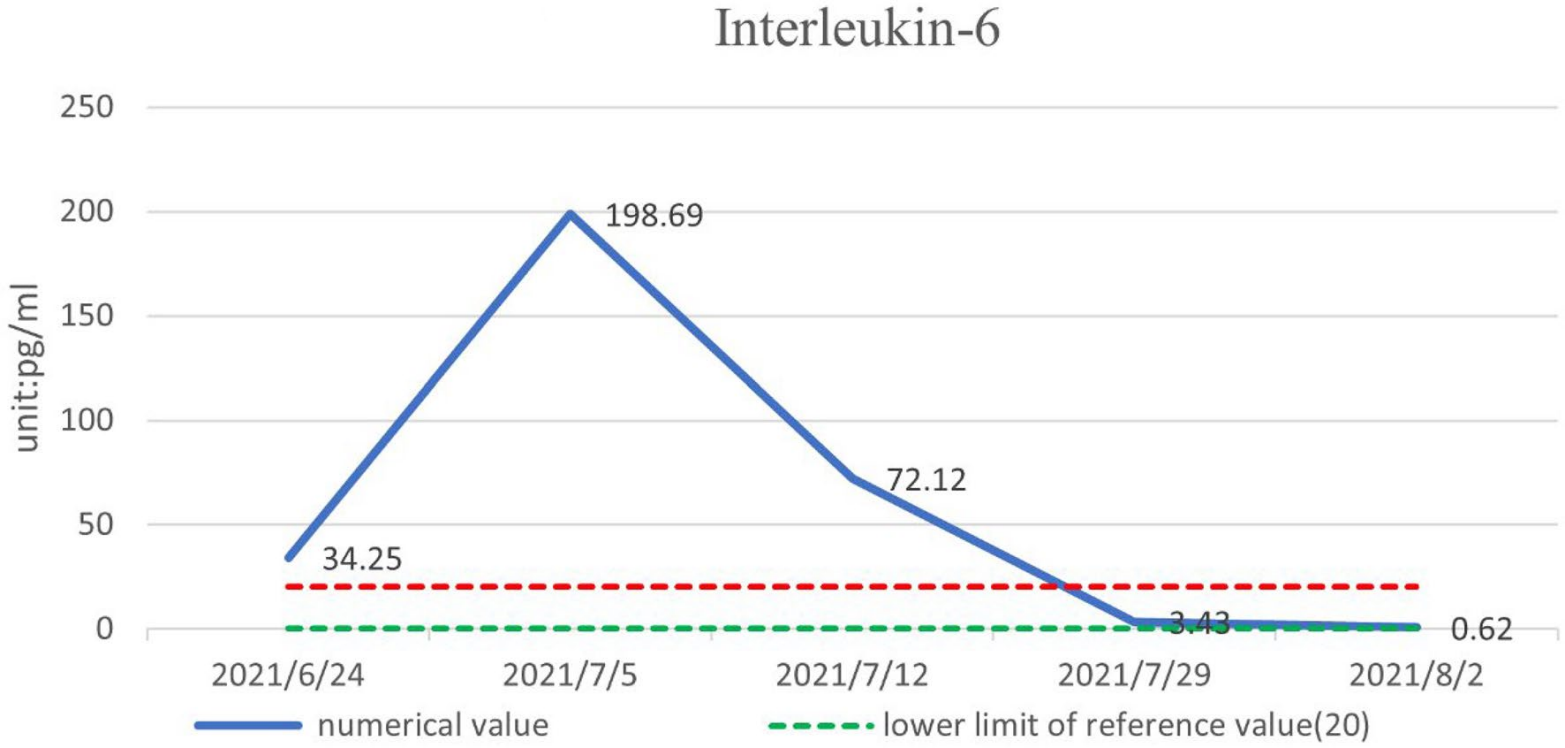
Inflammatory markers (IL-6) returned to normal after 1 month of meprednisolone and tozizumab injections

**Fig. 4:**
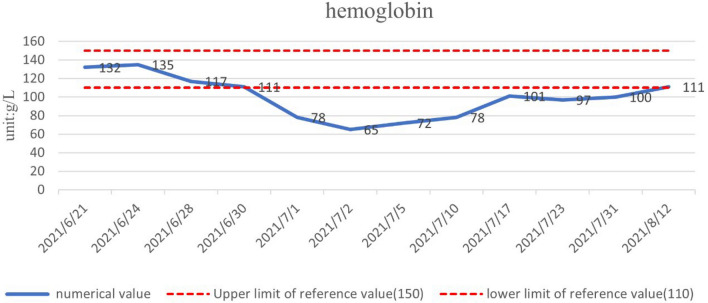
Hemoglobin returned to normal after 1 month of treatment with iron supplements such as folic acid

**Fig. 5:**
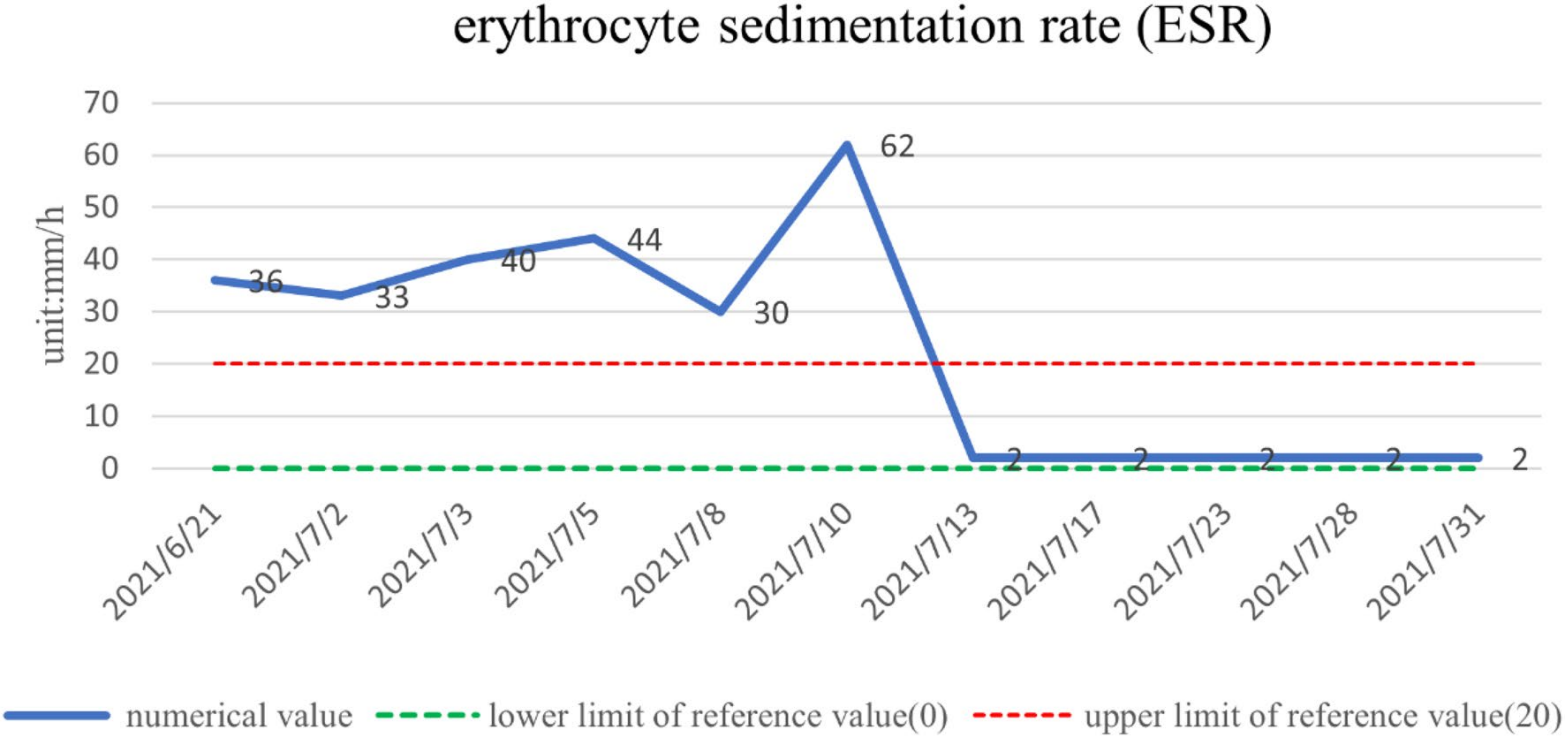
ESR returned to normal after 1 month of meprednisolone

**Fig. 6:**
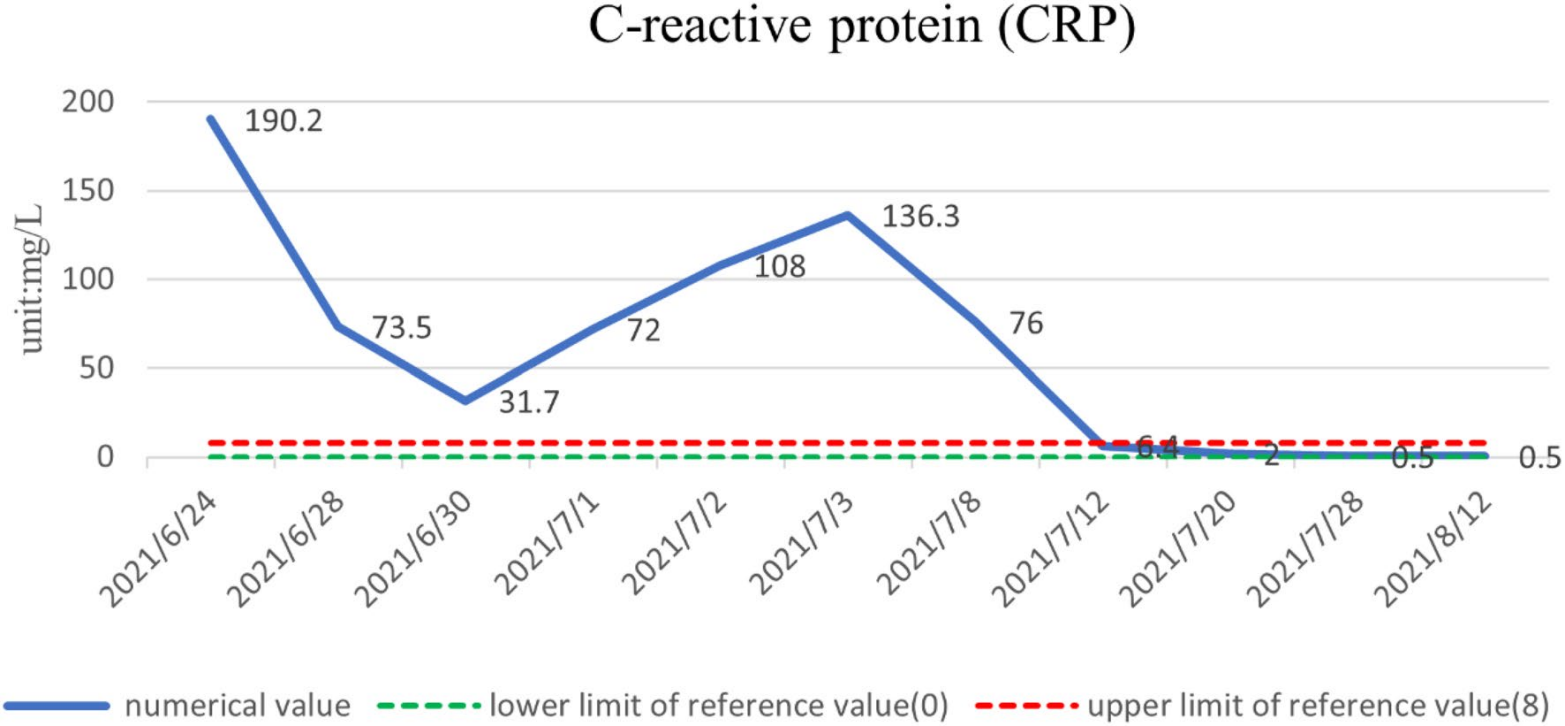
CPR returned to normal after 1 month of meprednisolone

The patient was treated with methylprednisolone at a starting dose of 80 mg/day for 13 days, tapered to 40 mg/day for 7 days, and then further reduced, with methylprednisolone tablets 32 mg/day slowly reduced to 20 mg/day for maintenance. The dose-reduction process lasted for 34 days. She also received anti-cytokine therapy with an IL-6 antagonist (tocilizumab injection). Her diabetes insipidus was treated with oral desmopressin. After 1 month of treatment, her pain score (Numerical Rating Scale) had decreased from 6 to 1 and her body temperature, electrolytes, D-dimer, inflammatory markers (ESR, CRP, IL-6), and urine volume were all normal. There was a marked increase in her hemoglobin levels. The objective indexes were thus significantly improved. A timeline of the clinical indicators (IL-6, hemoglobin, ESR, CRP) and interventions are summarized in Figs. 3, 4, 5, and 6.

## Discussion

Histiocytoses are rare disorders that are difficult to define because of their complex clinical manifestations and diverse and heterogeneous presentations. ECD is a form of systemic, non-Langerhans cell histiocytosis that was first described by Jakob Erdheim and William Chester in 1930. It is histologically characterized by the infiltration of foamy, lipid-laden, CD68(+) and CD1a(−) histiocytes. However, the pathogenesis of ECD is still uncertain. Previous studies have indicated roles for mutated oncogenes, including the nucleotide variant *BRAF*V600E and mutations in *MAP2K1*, *KRAS*, *NRAS*, *PIK3CA*, and C*SF1R*, inflammation involving cytokines and chemokines, clonal histiocytic proliferation, and immune-mediated mechanisms [9–13].

The current patient presented with abdominal pain and fever as the chief complaints. Imaging confirmed the cause of the abdominal pain as a splenic infarction. We subsequently detected multiple thromboses in the aortic arch and left subclavian artery, right side of the common femoral artery, and bilateral internal iliac arteries. Ischemic heart disease, heart failure, valvular disease, pericardial effusion, and conduction system abnormalities are common clinical manifestations of ECD, mostly attributed to involvement of the cardiovascular system. A review of patients with ECD who underwent CT or MRI found that 72/178 (40%) patients had cardiovascular involvement, of whom 75% had cardiac involvement [8]. Vascular manifestations include periaortic fibrosis, “coated aorta”, and renal artery involvement, with infiltration of the aorta and its branches being the most common. Another study in 24 patients with ECD found that most patients were male and the mean age at diagnosis was 58 years [7]. Pericardial involvement (13%), myocardial infiltration (25%), endocardial involvement (4%), valvular disease (17%), aortic/vascular disease (17%), conduction system infiltration (8%), and coronary artery disease (25%) were all present. The mortality was 17% after a median follow-up of 5.5 years.

Vascular involvement is rare in patients with ECD. Among all reported cases with arterial disease, including that affecting the coronary artery, cerebral artery, thoraco-abdominal aorta, subclavian artery, and renal artery, periarteritis is the main manifestation [14–19], In a 53-year-old woman with ECD, both the thoracic aorta and superior vena cava have been found to be involved [19]. A successful case [20] of use of the *MEK* inhibitor Cobimetinib in the treatment of multiple arterial thrombosis in a 71-year-old man with underlying diseases such as hypertension and myeloproliferative neoplasm has been reported. Importantly, a bone marrow biopsy of the patient showed essential thrombocythemia (TE) with JAK2 mutation, all of which are involved in thrombosis but not ECD itself. The myeloid lineage differentiation plays a central role in the pathogenesis of histiocytosis. Monocytes are myeloid-derived white blood cells, and monocyte subsets and ECD in a patient with severe vascular disease requiring liver transplantation have been reported [21]. The results indicated that a CD14++CD16− "classical monocyte" increase was associated with disease activity, thus suggesting that ECD is similar to chronic myelomonocytic leukemia (CMML). CD14+ monocyte expansion is associated with flares in ECD, independently of the monocyte count in histiocytosis. Unfortunately, the patient died despite receiving interleukin-1 receptor antagonist (Anakinra) and *MEK* inhibitor (Cobimetinib) treatment. Lymphocyte subgroup analysis was performed in our case, which showed normal T, B, NK, and CD16/56 cells. We did not detect CD14+ monocytes. However, we observed that our patient’s monocyte count was elevated before she was admitted to the hospital, accounting for as much as 1% of white blood cells. This result supports the point mentioned above. However, a retrospective study of 78 patients with ECD in France (60 men and 18 women) has found that hypoalphalipoproteinemia (HDL) and *BRAFV600E* mutation are major predictors of aortic infiltration in the ECD, and cardiovascular disease is detected in 84% of patients with ECD, thus suggesting that hypoalphalipoproteinemia in male ECD patients carrying the *BRAFV600E* mutation favors the formation of lipid-laden histiocytes, and the *BRAF* status and HDL phenotype are independent determinants of the aortic involvement in ECD [22]. In our case, the patient's blood lipids, including low density lipoprotein cholesterol (LDL-C), were slightly elevated (3.68 mmol/L, normal 1.31–1.37 mmol/L), but her HDL was low (0.68 mmol/L, normal 0.90–1.95 mmol/L). Interestingly, our patient was a woman negative for *BRAFV600E* mutation.

Another important feature in our patient was multiple thrombosis. The formation of thrombosis requires three conditions: first, vascular endothelial injury, second, blood flow state change, and third, increased blood coagulation. Arterial biopsy has confirmed that perivascular infiltration and fibrosis are the main pathological basis of vascular lesions in patients with ECD [23]. In a case report of a patient with cerebral microvascular ECD, the pathological results of multiple brain biopsies and vascular biopsies suggested chronic meningoencephalitis and perivasculitis; eventually, the patient died because of persistent recurrent fever and neurological deterioration [24]. Another biopsy of the mesenteric artery has indicated adventitia fibrosis with normal intima and media, without atherosclerosis or vasculitis [21]. In that patient, the arterial biopsy of the superficial circumscribed iliac vein of the left lower limb revealed only slight endothelial thickening, and no neutrophils, lymphocyte infiltration, or vasculitis was found. Therefore, the cause of multiple thrombus formation cannot be explained by perivascular infiltration and fibrosis, and the pathological mechanism of multiple thrombus formation needs further study.

A review of the above literature indicates that extensive vascular involvement, especially of the large vessels, such as the thoraco-abdominal aorta and mesenteric arteries, is associated with a high mortality rate and poor prognosis. Therefore, this case highlights the need for vascular and cardiac surgeons to be aware of the possibility of ECD in the differential diagnosis of polythrombosis and provide early intervention.

ECD potentially affects any organ, but bone is considered to be a major target organ, characterized by symmetrical long bone diaphyseal and metaphyseal osteosclerosis, with up to 96% of patients having bone changes detected by imaging, usually around the knees and ankles.

However, ECD can also occur in other bones. A case of simple destruction of the right second rib diagnosed as ECD was recently reported from Nepal [25]. The main radiographic findings of the disease include bilateral, symmetric diametaphyseal sclerosis of the long bones, which manifest on MRI as extensive replacement of the fatty marrow by low signal on T1WI, heterogeneous signal on T2WI/STIR, and enhancement after gadolinium injection [26–28]. In addition, 15% of patients have osteosclerosis with a mixed pattern of lytic and sclerotic lesions as rare skeletal manifestations of ECD [27]. Even children may present with isolated skeletal involvement in the form of multiple osteolytic lesions [30]. Typical symptoms include bone pain [31]; however, despite the high prevalence of skeletal infiltration, 60% of patients are asymptomatic [5]. Although imaging in the current patient suggested diffuse osteodestruction of both knees, the patient had no complaints of knee pain. Patients without bone pain as a major complaint should thus also undergo imaging examinations to detect possible bone involvement.

The CNS is also commonly affected by ECD. The 5-year survival rate of patients with ECD is 68% [32], with the presence of cardiovascular and CNS disease contributing to the poor prognosis [33]. CNS abnormalities have been reported in half of all ECD cases [34]. Diabetes insipidus is also common in cases of panhypopituitarism, and ECD patients with central diabetes showed pituitary stalk thickening, accompanied by gonadotropin deficiency [35]. The current patient’s diagnosis was delayed for 3 years. This patient also had diabetes insipidus with a daily urine volume > 5000 mL. Fortunately, we made a positive diagnosis and administered effective treatment, and the patient’s daily urine volume was subsequently maintained at around 2000 mL, with a great improvement in her quality of life.

The clinical spectrum of ECD is diverse and the treatment thus needs to vary accordingly, including local and systemic treatment. Despite the availability of new drugs, there is currently no consensus on the treatment for ECD, mainly due to the lack of controlled studies, which are difficult to perform because of the rarity of the disease. However, interferon (IFN)-α has been identified as an independent predictor of survival [29], B-Raf inhibitors are recommended as first-line treatment for patients with severe organ involvement and *BRAF*V600E [36, 37], and B-Raf inhibitors (vemurafenib or dabrafenib), pegylated-IFN-α/IFN-α, anakinra, imatinib, infliximab, corticosteroids, and radiotherapy have been considered as second-line treatments [38–40]. Patients with ECD showed strong systemic immune activation, involving IFN-a, IL-1/IL1-RA, IL-6, IL-12, and MCP-1, in agreement with the systemic immune Th-1-oriented disturbance associated with the disease. ECD has also been suggested to be an inflammatory myeloid neoplasia. Between 57 and 75% of patients with ECD carry the *BRAFV600E* mutation, an activating mutation of the proto-oncogene *BRAF*. More than 50 patients worldwide with BRAF mutations and severe multisystem refractory ECD (sometimes accompanied by Langerhans cell histiocytosis, LCH) have received vemurafenib. The report has also suggested that ECD should be redefined as an inflammatory myeloid neoplasia, because it is based on a background of chronic inflammation, mutations in the mitogen-activated protein kinase (MAPK) pathway are found in ECD and LCH [41], and deregulated activation of the MAPK pathway due to oncogenic mutations in the *BRAF*, *NRAS*, *PIK3CA*, and *MAP2K1* genes is central to the pathogenesis of ECD [40]. Therefore, small molecule inhibitors of *BRAFV600E* (vemurafenib) or MEK (Cobimetinib, trametinib) have shown promise in ECD treatment [43].

In addition, some cases of ECD do not have targeted kinase mutations, and thus treatment options are lacking. Interleukin (IL-6) is a pleiotropic cytokine involved in regulating the immune response and bone metabolism; it is produced in abundance by foam tissue cells in ECD lesions. On this theoretical basis, an open-label, single-arm, phase II, prospective, pilot study of tocilizumab in ECD has been conducted (ClinicalTrials.gov NCT01727206; Eudra-CT 2012-003151-11). This study included three patients with contraindications or a lack of response to IFN-a therapy, and evaluated the clinical and radiologic changes, as well as the modulation of pro-inflammatory mediators. The BRAFV600E mutation was detected in all cases. The treatment results showed that all three patients had cardiovascular system involvement, mainly manifested as heart failure and symptomatic pericardial effusion. Cardiac MRI performed at 28 weeks showed improved diastolic and systolic function (LFEV 45%), a 50% decrease in pericardial effusion, and markedly diminished pseudo tumoral infiltration of the right atrium, from 12 to 7 mm in diameter. One patient with nervous system involvement did not respond to treatment. Quality of life improved in two patients, and fever, fatigue, and pain disappeared in all patients [44].

All genetic tests were negative in the current case, but IL-6 levels were significantly elevated. We therefore administered methylprednisolone to inhibit the immune inflammatory response and anti-cytokine therapy with the IL-6 antagonist tocilizumab. Of note, glucocorticoids are present throughout the patient's treatment. When patients are diagnosed with ECD, the patient's body inflammation index increases significantly. After many negative blood culture results and multiple courses of effective broad-spectrum antibiotics failed, we decided to administer high-dose glucocorticoids, which resulted in a gradual reduction of her symptoms. Later, after combined treatment with an IL-6 antagonist, the objective data indicated a satisfactory curative effect, with decreases in various inflammatory indexes and control of her clinical symptoms, suggesting that hormonotherapy and cytokine/chemokine therapy may be potential novel therapeutic strategies for patients with ECD without gene mutations.

Here we present the report of a patient with ECD with multiple thromboses, embolism, and organ involvement as the main presenting characteristics. Our experience suggests that, if no obvious positive signs are found after routine screening for thrombi in the thoracic aorta and other arteries, especially in patients with damage to other systems, X-ray examination of the joints should be performed for preliminary screening to provide further clues and avoid the risk of a missed diagnosis. Biopsy of the lesion site is the ultimate method for determining the nature of the lesion.

The results of this study indicate the need for tests and examinations to determine the presence or absence of ECD in patients with unexplained arterial thrombosis.
